# Parvovirus B19-associated fat embolism syndrome in sickle cell disease: A report of two cases

**DOI:** 10.1016/j.clinme.2026.100587

**Published:** 2026-04-29

**Authors:** Anna Corby, Jessica Lubel, Sara Stuart-Smith, Arne de Kreuk, Rachel Kesse-Adu, Temi Lampejo

**Affiliations:** a Department of Haematology, King’s College Hospital NHS Foundation Trust, United Kingdom; b Department of Radiology, King’s College Hospital NHS Foundation Trust, United Kingdom; c Department of Haematology, Guy’s and St. Thomas’ NHS Foundation Trust, United Kingdom; d Department of Infection Sciences, King’s College Hospital NHS Foundation Trust, United Kingdom

**Keywords:** Fat embolism transfusion, Red cell exchange, Plasma exchange, PLEX, TPE, ECMO, Parvovirus

## Abstract

Fat embolism syndrome (FES) is a rare but likely underdiagnosed complication of sickle cell disease (SCD) characterised by multi- or single-organ involvement secondary to embolism of fat and/or necrotic bone marrow. It is one of the most devastating acute complications of SCD and historically the diagnosis has often been made on the basis of post-mortem findings. Literature on FES in SCD is scarce, although most reported cases are individuals with non-HbSS genotypes for reasons which have not yet been fully elucidated. Even fewer data exist regarding the potential association with parvovirus B19 (B19V) and the mechanisms by which B19V infection could trigger FES in SCD. Management is largely supportive, with early intensive care team involvement for close monitoring and potential organ support. Emerging evidence, albeit limited, suggests that early exchange transfusion has an important role in improving clinical outcomes. We describe two life-threatening cases of FES in patients with SCD of differing genotypes, who required intensive care unit admission but who both recovered with supportive management and early exchange transfusion.

## Highlights

• Fat embolism syndrome should be considered in patients with sickle cell disease (all genotypes) who develop severe acute neurological and/or respiratory manifestations.
• Haematological or biochemical abnormalities may include cytopenias (particularly marked thrombocytopenia and/or anaemia), raised lactate dehydrogenase, raised alkaline phosphatase, raised C-reactive protein (>100 mg/L), profoundly elevated ferritin (>10,000 μg/L), and a leucoerythoblastic picture on a blood film.
• Parvovirus B19 infection should considered as a potential trigger (and be investigated for) in cases of sickle cell disease-associated fat embolism syndrome.
• Early exchange transfusion including plasma exchange is associated with improved outcomes.

## Introduction

Fat embolism syndrome (FES) is a devastating condition affecting individuals with sickle cell disease (SCD), which, along with parvovirus B19 (B19V) infection as a potential precipitant, is largely under-recognised. Sadly, the diagnosis of FES has often been made at post-mortem.^1^ We describe two severe cases of FES in SCD occurring in association with acute B19V infection.

## Case one

Our first patient is a 23-year-old woman with known SCD (HbS/Beta(+) thalassaemia genotype), for which she had one medical presentation per year on average and had previously received only a single red cell transfusion following childbirth. She had no other significant medical history. She presented to the hospital emergency department (ED) with an acute-onset chest and back pain. Her pain settled rapidly in the ED with analgesia and she was discharged home, but re‐presented later that day with more widespread pain, most prominent in her lower limbs. All her initial observations were normal. The abnormalities on her admission blood tests were a microcytic anaemia, with haemoglobin (Hb) of 102 g/L (normal range 120–150 g/L), a low reticulocyte count of 6 × 10^9^/L, (normal range 50–150 × 10^9^/L), a marginally elevated lactate dehydrogenase (LDH) at 232 U/L (normal range 135–214 U/L) and an elevated C-reactive protein (CRP) of 349 mg/L (normal range <5 mg/L). Table 1 shows the evolution of the haematological and biochemical parameters during the hospital admission. She was admitted to a haematology ward, where she received opiate analgesia and broad-spectrum antibiotics. A blood film showed a leucoerythroblastic picture. B19V DNA was detected (1.2 × 10^6^ IU/mL, genotype 1) in an admission blood sample and B19V IgM and IgG were both positive consistent with an acute/recent B19V infection. 2 weeks prior, her 7-year-old son had a confirmed B19V infection.

**Table 1:** Haematological and biochemical blood test results for both patients during their hospital admission.

	Haematological or biochemical parameter	Day of admission	Day 3	Day 7	Day 14	Day 21
**Patient 1 (female)**	Hb (NR 120–150 g/L)	**102**	**84**→**54**	**81**	**76**	**99**
	Reticulocyte count (NR 50–150 × 10^9^/L)	**6**	**13**	**27**	93	-
	Platelet count (NR 150–400 × 10^9^/L)	221	**28**	**56**	**47**	**82**
	WBC count (NR 4–11 × 10^9^/L)	8.3	**12.7**^a^	**3.4**	**3.9**	**12.5**
	ALP (NR 35–129 IU/L)	75	**472**	**197**	**227**	**209**
	ALT (NR 4–59 IU/L)	14	47	33	39	**251**
	eGFR (NR >90 mL/min)	>90	**86**	>90	>90	>90
	LDH (NR 135–214 U/L)	**232**	**3884**	**2220**	**853**	-
	Ferritin (NR 15–200 μg/L)	-	**35,039**	**3043**	-	-
	CRP (NR <5 mg/L)	**349**	**328**	**510**	**126**	**23**
**Patient 2 (male)**	Hb (NR 120–150 g/L)	**64**	**79**	**96**	**87**	
	Reticulocyte count (NR 50–150 × 10^9^/L)	**29**	**182**	**296**	**213**	
	Platelet count (NR 150–400 × 10^9^/L)	**103**→**59**	**94**	247	357	
	WBC count (NR 4–11 × 10^9^/L)	**28.9**^b^	**22.1**	**15.9**	5.8	
	ALP (NR 35–129 IU/L)	**397**	**229**	**419**	**316**	
	AST (NR 0–45 IU/L)	**81**	**137**	**139**	**86**	
	eGFR (NR >90 mL/min)	>90	**61**	>90	>90	
	LDH (NR 135–214 U/L)	**283**	-	-	-	
	Ferritin (NR 30–400 μg/L)	**36,240**	**2875**	-	**1491**	
	CRP (NR <5 mg/L)	**175**	**112**	**101**	**17**	

Abnormal results are highlighted in bold.

Abbreviations: Hb, Haemoglobin; NR, normal range; WBC, white blood cell; ALP, alkaline phosphatase; ALT, alanine aminotransferase; eGFR, estimated glomerular filtration rate; LDH, lactate dehydrogenase; CRP, C-reactive protein.

a Elevated neutrophil and lymphocyte counts.

b Elevated neutrophil, lymphocyte and basophil counts.

**Fig. 1:**
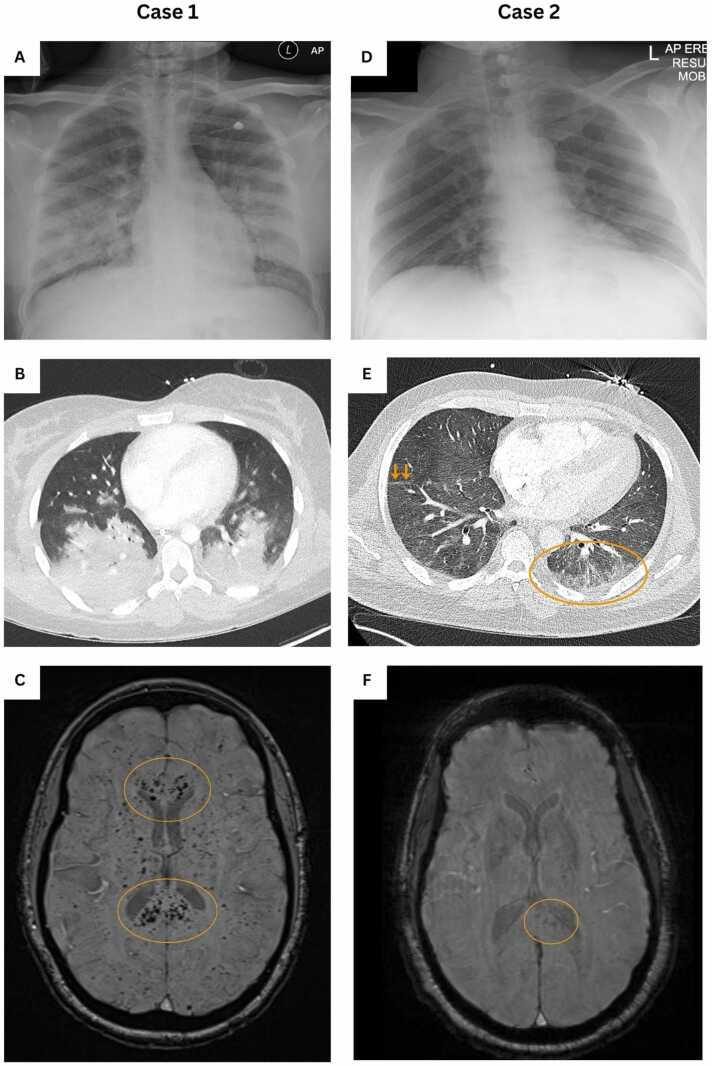
Imaging of the chest and brain for the patients in cases 1 and 2. Case 1: (A) Chest radiograph: bilateral mid-zone airspace opacification. (B) Axial computed tomography (CT) images of the thorax, lung window: bilateral severe consolidation. (C) Axial magnetic resonance (MR) images of the brain, susceptibility weighted imaging (SWI) sequence: innumerable punctate foci of susceptibility within both cerebral hemispheres, most densely within the corpus callosum (circled). Case 2: (D) Chest radiograph: no focal consolidation, collapse, or effusion. (E) Axial CT images of the thorax, lung window: ground-glass opacification in the left lower lobe (circled) and linear atelectasis (arrows). (F) Axial MR images of the brain, SWI sequence, degraded by motion artefact: punctate foci of susceptibility, less diffuse than in Case 1, most evident in the splenium of the corpus callosum, to the left of midline (circled).

Approximately 36 h after admission, she acutely deteriorated with profound hypoxia, tachypnoea, tachycardia, agitation and abnormal posturing. At this stage, her cytopenias were worsening; Hb 84 g/L from a baseline of ∼125 g/L, and platelets 28 × 10^9^/L (normal range 150–400 × 10^9^/L) from a baseline of 221 × 10^9^/L. CRP remained elevated at 328 mg/L. Liver function tests (LFTs) were abnormal with alkaline phosphatase 472 IU/L (normal range 35–129 IU/L) but a normal alanine aminotransferase (ALT). Her estimated glomerular filtration rate (eGFR) was slightly reduced at 86 mL/min. There was a marked increase in LDH to 3884 U/L and ferritin to 35,039 μg/L (normal range 30–400 µg/L). She required intubation and transfer to the intensive care unit (ICU). A chest radiograph showed bilateral diffuse infiltrates and a computed tomography pulmonary angiogram (CTPA) scan identified no pulmonary embolus, but showed severe bilateral consolidation (Fig. 1). Antimicrobial cover was escalated from ceftriaxone to meropenem. ADAMTS13 enzyme activity was within normal limits, therefore thrombotic thrombocytopenic purpura (TTP) was deemed unlikely. A CT head did not identify any acute abnormalities, but brain magnetic resonance imaging (MRI) identified innumerable punctate foci of susceptibility in keeping with microhaemorrhages, in a distribution consistent with FES. The cytopenias worsened. Emergency manual red cell exchange transfusion was carried out on day 3. Her respiratory function continued to deteriorate, and in view of refractory hypoxaemia, she was placed on venovenous extracorporeal membrane oxygenation (VV-ECMO) for 7 days.

Aiming to suppress viral replication, intravenous immunoglobulin (IVIG) was administered on day 14 in view of sustained cytopenias, which were presumed due to non-resolving B19V infection. She spent 24 days in the ICU before being stepped down to a haematology ward, where she required regular dietetic input, physiotherapy, occupational therapy, and speech and language therapy. She was discharged home 5 weeks after admission and, at clinic review 3 months later, had significantly improved although she reported memory impairment, low mood and intermittent headaches.

## Case two

A 44-year-old man with SCD (HbSC) presented with a <24 h history of chest, bilateral shoulder and bilateral hip pain and fever. His initial Hb was 121 g/L (with a low reticulocyte count of 4 × 10^9^/L) and as his pain rapidly settled with analgesia, it was agreed to manage him via the ambulatory care pathway. He re-presented 2 days later with confusion, breathlessness and widespread pain. He was afebrile but tachycardic (heart rate 150 bpm) and desaturating, requiring 4 L/min of supplementary oxygen. On examination he was confused (Glasgow Coma Scale 14/15), visibly in distressed and tachypnoeic, but his chest was clear on auscultation. An admission chest radiograph was normal. A CTPA identified no evidence of pulmonary emboli, but bibasal atelectasis and ground-glass opacification were noted (Fig. 1). A CT head was normal. Abnormalities on admission blood tests included anaemia (Hb 65 g/L, usual baseline 120 g/L), a low reticulocyte count of 29 × 10^9^/L (usual baseline 150 × 10^9^/L), a raised white cell count of 28.9 × 10^9^/L (normal range 4–11 × 10^9^/L), a raised LDH of 283 U/L (from a baseline of 140 U/L), a raised ALP of 397 IU/L (previously normal at 103 IU/L 2 days earlier), a markedly elevated ferritin of 36,240 μg/L, and a raised CRP of 175 mg/L (Table 1). His platelet count rapidly fell shortly after being admitted to 59 × 10^9^/L from a usual baseline of 120 × 10^9^/L. His renal function was normal on admission, but also transiently deteriorated shortly after. A leucoerythoblastic picture was seen on blood film. He received opiate analgesia, supplementary oxygen, intravenous fluids, broad-spectrum antibiotics (amoxicillin–clavulanate and doxycycline for a possible chest infection) and was transfused 3 units of red cells. An admission blood sample was positive for B19V IgM, IgG and B19V DNA at a high level (3 × 10^7^ IU/mL), consistent with an acute B19V infection, and his next of kin reported a recent B19V infection in his daughter.

On day 2 of admission, he received full automated red cell exchange transfusion. He was admitted to the ICU the following day with worsening confusion and agitation requiring sedation. He continued to have fluctuating supplementary oxygen requirements (up to 60% FiO2 via Venturi mask), but did not require intubation. Antibiotics were escalated to piperacillin–tazobactam. An MRI brain showed foci of susceptibility predominantly in the white matter, in keeping with microhaemorrhages and consistent with FES. Given the lack of clinical improvement on his first day in the ICU and the clinico-radiological findings of FES, that day he underwent therapeutic plasma exchange (PLEX) with replacement of 1.0 plasma volumes using solvent detergent-treated pooled human plasma (Octaplas) as the replacement fluid. PLEX was continued daily; he showed signs of clinical improvement after 3 consecutive days of PLEX and a decision was made to complete 5 days in total. His cognition improved, his supplementary oxygen requirement resolved and he was deemed well enough to be stepped down to the haematology ward after 6 days on the ICU. His reticulocyte count also significantly improved. He continued to improve on the ward with intensive physiotherapy, occupational therapy, and speech and language therapy. He was discharged home 18 days after admission. At clinic review 1 month later, he was well with no ongoing cognitive issues and had returned to work, although he did report tiredness on exertion and impaired memory.

## Discussion

FES occurs due to the release of globules of fat into the systemic circulation. It is most commonly described in the context of trauma and typically has haematological, respiratory, neurological and/or cutaneous manifestations. The Gurd and Wilson criteria are the most widely recognised diagnostic criteria for FES and although both our patients met the criteria for a diagnosis of FES on the basis of this tool, it was primarily designed to diagnose FES in the context of bone fractures.^2^ Non-traumatic FES, for which established diagnostic criteria are lacking, is considered to be rare and is associated with bone marrow necrosis.^3^, ^4^ A blood film typically shows a leucoerythroblastic picture and a bone marrow trephine may show disruption of normal marrow architecture with loss of fat spaces. Extensive bone marrow necrosis can result in pancytopenia. During a SCD vaso-occlusive crisis, fat and haematopoietic tissue entering the systemic circulation can embolise to the lungs, causing an acute chest syndrome, whereas in FES, the extensive bone marrow necrosis that occurs can result in the release large quantities of fat/necrotic marrow into the peripheral circulation, affecting multiple organs including the chest, with clinical features in keeping with multi-organ dysfunction.^1^ Although initially presenting with an apparent acute pain crisis, both of our patients rapidly deteriorated and progressed to severe neurological and respiratory impairment. FES may be misdiagnosed as an acute chest syndrome, a stroke or even TTP. However, FES should be considered in a previously well patient with SCD presenting with what initially appears to be an uncomplicated pain crisis, or in our experience, an unusually severe pain crisis, followed by rapid deterioration, with progressive thrombocytopenia, raised LDH, raised ALP, raised CRP and profoundly elevated ferritin (>10,000 µg/L). In the largest single-centre case series of FES in SCD comprising 19 patients (four of whom had evidence of recent B19V infection), all 19 patients had LDH >1,000 U/L, ALP >200 U/L, CRP >100 mg/L and ferritin >1,000 μg/L, and 16/19 (84%) had ferritin >10,000 µg/L.^5^

In terms of cerebral involvement in FES, fat droplets are considered to initially enter the pulmonary circulation and then either pass through an arteriovenous shunt, ie patent foramen ovale (present in approximately 25% of the general population) or travel directly through the pulmonary capillary bed into the systemic circulation, subsequently reaching the brain.^6^ Although the morphology and distribution of the microhaemorrhages seen on the MRI brain images of both our patients were particularly characteristic for FES, of note is that ECMO-related thrombotic microemboli, critical illness-associated cerebral microbleeds and diffuse axonal injury (typically occurring in the context of trauma rather than in SCD) can have overlapping features on MRI, albeit often distinguishable with the appropriate radiological expertise and relevant clinical context.^7^, ^8^ Recently, Nasiri *et al* developed a novel diagnostic scoring system for FES in SCD based on a combination of clinical, haematological, biochemical and radiological parameters.^9^ Their proposed scoring system, which was developed following a comprehensive review of published cases and represents the first evidence-based diagnostic tool specifically for FES in SCD, awaits prospective validation and evaluation of its potential clinical utility.

Various hypothetical mechanisms by which B19V could trigger bone marrow necrosis have been proposed, including direct cytotoxic effects, immune-mediated damage, and B19V induced endothelial injury.^10^ Interestingly, our cases occurred during periods of significantly increased B19V circulation, and both had a child recently diagnosed with a B19V infection. Our cases highlight the importance of increased vigilance for complications of SCD during periods of heightened community B19V circulation. Neither patient developed a B19V-associated rash.

In the largest systematic review of FES in SCD by Tsitsikas and colleagues, 58 cases were identified, of which 10 patients had a documented recent B19V infection (although a negative B19V result was recorded in only four of the 58 cases).^4^ A preceding non-specific viral illness was recorded in 10 patients, none of whom had documented B19V testing. Interestingly, and for reasons for which are not understood, most cases of FES have been associated with ‘milder’ forms of SCD/non-HbSS genotypes (only 19% had the HbSS genotype in their review), and both of our patients had non-HbSS genotypes. In addition to being a cornerstone of treatment for SCD, hydroxycarbamide (a potent inhibitor of DNA synthesis targeting the ribonucleotide reductase enzyme) has been shown to inhibit B19V replication *in vitro* and observational studies suggest a protective effect of hydroxycarbamide against severe human B19V infection and its associated complications in patients with SCD.^11^, ^12^ Whether this is a factor in why FES has been more frequently observed in non-HbSS genotypes, given that hydroxycarbamide is typically reserved for those with the HbSS genotype, remains to be elucidated.

In the review by Tsitsikas *et al*, 37 of the 58 (64%) patients with FES died; mortality was 91% without transfusion, 61% with top-up transfusion and 29% with exchange transfusion.^4^ Post-mortem examinations of patients who have died from SCD-associated FES have revealed fat emboli in multiple organs, most commonly including the lungs, brain, heart and kidneys.^13^ Early exchange transfusion is important in reducing mortality and evidence is now also emerging to suggest further improvements in outcomes with the sequential use of PLEX following red cell exchange.^14^, ^15^ Therefore, in patients who do not rapidly respond to initial red cell exchange transfusion, prompt initiation of PLEX should be strongly considered, given the increasing body of evidence to support this therapeutic strategy in improving both survival and neuro-cognitive outcomes.^16^ In view of the rapidly evolving nature of the syndrome, some experts even suggest considering pre-emptive exchange transfusion, prior to any evidence of organ failure, in high-risk patients, although determining who is most likely to benefit from this approach is currently challenging.^17^

There is no evidence to support routine empirical use of IVIG in SCD-associated FES. However, there is some suggestion mainly from case series that IVIG may help in controlling B19V replication, particularly in severe and/or prolonged B19V infection not controlled by host immune responses, for example due to immunosuppression.^11^ Given the persisting cytopenias observed in our first patient, potentially driven by ongoing B19V replication which specifically targets erythroid progenitor cells, she received IVIG. She survived following a period VV-ECMO on the ICU. Although only limited data are available, selective use of ECMO was associated with a strong trend towards improved survival in a select cohort of adults with SCD who developed acute chest syndrome.^18^

## Conclusion

We report two life-threatening cases of FES in SCD in association with B19V infection. Although further reports have been emerging, literature remains relatively scarce on this likely underdiagnosed complication of SCD, and greater awareness is needed, including the association with B19V infection.^1^, ^10^, ^19^ FES should be considered in patients with SCD who develop acute respiratory and/or neurological decline in the presence of rapidly worsening cytopenias. Early exchange transfusion incorporating PLEX, supportive care with early ICU team involvement and intensive rehabilitation are important in improving outcomes.

## CRediT authorship contribution statement

**Jessica Lubel:** Writing – review & editing, Writing – original draft, Investigation. **Sara Stuart-Smith:** Writing – review & editing, Supervision, Investigation. **Anna Corby:** Writing – review & editing, Investigation. **Temi Lampejo:** Writing – review & editing, Writing – original draft, Supervision, Investigation, Conceptualization. **Arne de Kreuk:** Writing – review & editing, Supervision, Investigation. **Rachel Kesse-Adu:** Writing – review & editing, Supervision, Investigation.

## Consent for publication

Written consent was obtained from both patients included in this manuscript to publish, including the use of anonymised images.

## Funding

This research did not receive any specific grant from funding agencies in the public, commercial or not-for-profit sectors.

## Declaration of competing interest

The authors declare that they have no known competing financial interests or personal relationships that could have appeared to influence the work reported in this paper.
